# 3D Woven Textile Structural Polymer Composites: Effect of Resin Processing Parameters on Mechanical Performance

**DOI:** 10.3390/polym14061134

**Published:** 2022-03-11

**Authors:** Rajesh Kumar Mishra, Michal Petru, Bijoya Kumar Behera, Promoda Kumar Behera

**Affiliations:** 1 Department of Material Science and Manufacturing Technology, Faculty of Engineering, Czech University of Life Sciences Prague, Kamycka 129, 16500 Prague, Czech Republic; 2 Department of Machinery Construction, Faculty of Mechanical Engineering, Technical University of Liberec, Studentska 1402/2, 46117 Liberec, Czech Republic; michal.petru@tul.cz (M.P.); promodabehera@gmail.com (P.K.B.); 3 Department of Textile & Fiber Engineering, Indian Institute of Technology Delhi, New Delhi 110016, India; behera@textile.iitd.ac.in

**Keywords:** textile structural composite, epoxy resin, add-on (%), amount of hardener (%), curing temperature, curing time, molding pressure, mechanical properties

## Abstract

This work presents the manufacture of polymer composites using 3D woven structures (orthogonal, angle interlock and warp interlock) with glass multifilament tows and epoxy as the resin. The mechanical properties were analyzed by varying the processing parameters, namely, add-on percentage, amount of hardener, curing time, curing temperature and molding pressure, at four different levels during the composite fabrication for three different 3D woven structures. The mechanical properties of composites are affected by resin infusion or resin impregnation. Resin infusion depends on many processing conditions (temperature, pressure, viscosity and molding time), the structure of the reinforcement and the compatibility of the resin with the reinforcement. The samples were tested for tensile strength, tensile modulus, impact resistance and flexural strength. Optimal process parameters were identified for different 3D-woven-structure-based composites for obtaining optimal results for tensile strength, tensile modulus, impact resistance and flexural strength. The tensile strength, elongation at break and tensile modulus were found to be at a maximum for the angle interlock structure among the various 3D woven composites. A composition of 55% matrix (including 12% of hardener added) and 45% fiber were found to be optimal for the tensile and impact performance of 3D woven glass–epoxy composites. A curing temperature of about 140 °C seemed to be optimal for glass–epoxy composites. Increasing the molding pressure up to 12 bar helped with better penetration of the resin, resulting in higher tensile strength, modulus and impact performance. The optimal conditions for the best flexural performance in 3D woven glass–epoxy composites were 12% hardener, 140 °C curing temperature, 900 s curing time and 12 bar molding pressure.

## 1. Introduction

Composites can be defined as a selected combination of different materials formed with a specific geometrical structure and with a specific external shape or form. These are materials created of two or more different components (or phases) that are distinguishable and separated by an interface. One of these dissimilar materials is called the reinforcement and the other component is called the matrix. A textile structural composite is a polymer composite that is defined as the combination of a resin system with a textile-fiber-, yarn- or fabric-based reinforcement system. Textile-fabric-reinforced composites may be flexible or quite rigid [1,2]. Having a non-crimp 3D fabric as a composite reinforcement is obviously beneficial because of the significantly higher in-plane stiffness and strength. A single-layer preform or a few layers of such a preform combined with an advanced, automatically controlled closed-mold resin infusion system allows for avoiding a lot of flaws and irregularities. The fabric structural parameters, design and preparation of preforms, etc., influence the resin impregnation [3].

A single-layer, relatively thick, 3D woven composite is often better than a much thinner single layer 2D woven composite or a 2D woven laminate that does not match the thickness of the 3D woven composite. Quite often, composites made using vacuum-assisted resin transfer molding (VARTM) are compared to those made via compression molding under much higher pressure (resulting in significantly higher fiber volume fraction). Furthermore, the in-plane fiber architecture of the compared 3D weave and 2D weave composites may be significantly different. Moreover, factors such as the resin system, composite processing cycle and environmental conditions may be different. Any of these factors would produce a significantly quantifiable effect on the resulting properties of composite materials [4,5,6].

The mechanical properties of a laminated fiber-reinforced composite suffers significantly by a void content exceeding 2%. The presence of voids in a 3D reinforced composite materials, owing to the reinforcement in the thickness direction, would not affect their properties as much as in unidirectional or 2D composites, such as laminates. For woven fabrics, the out-of-plane orientations of the warp and weft yarns at their cross-over points are taken into account in an approximate way by representing the curved yarns as a set of straight yarn segments [7,8,9].

Three-dimensional woven fabrics exhibit a higher thickness and superior inter-laminar properties compared to laminated 2D woven fabrics because of their integrated internal geometry. While traditional laminated composites constitute the vast majority of applications, 3D woven composites have certain advantages over laminates because of the controlled orientation of the fibers in all three directions. Three-dimensional woven composites have superior through-thickness strength, stiffness and thermal conductivity compared to conventional 2D laminated composites. In addition, the interlocking nature of a 3D woven fiber preform leads to superior damage tolerance and fatigue behavior [10,11,12]. Liquid composite molding (LCM) processes, such as resin transfer molding (RTM), are identified as one of the potentially most advantageous manufacturing techniques. Textile preforms have several length scales, starting from the individual fibers making up the fiber bundles, which are woven or stitched together to create the fabric [13,14]. In effect, fabric reinforcement exhibits heterogeneous behavior, whereby the fiber bundles are porous entities with free open spaces between them.

The purpose of the polymer matrix is to bind the fibers or reinforcement together by virtue of its cohesive and adhesive characteristics, transfer the load to the reinforcing fibers and protect them from environments and handling. When unsaturated polyester resins are cured, the monomer reacts with the unsaturated sites on the polymer, converting it to a solid thermoset structure. The ester groups of vinyl ester are susceptible to water degradation via hydrolysis, which means that vinyl esters exhibit better resistance to water and many other chemicals than their polyester counterparts and are frequently found in applications such as pipelines and chemical storage tanks. The viscosity of the resin/hardener combinations limits the selection of a resin with a viscosity suited to application equipment and fabric weave and weight. A hardener that provides adequate curing time based on the ambient temperature in the environment must be selected [15,16,17,18].

The mechanical properties of polymer composites can be affected by resin infusion or resin impregnation. Resin infusion depends on many parameters, such as the processing conditions (temperature, pressure, viscosity and molding time), the structure of the reinforcement and the compatibility of the resin with the reinforcement. As viscosity increases, the resin infusion decreases, and as applied pressure increases, the infusion also increases. It reduces the add-on of the resin/matrix and removes air from inside the structure to reduce the void formation. It is therefore essential to optimize these parameters. The curing temperature also plays a very important role in resin impregnation, as it may cause degradation of the resin and fiber. The curing temperature and time needs to be optimized to achieve a requisite level of impregnation [19,20,21,22,23,24,25].

The principal objectives of this research were to produce 3D woven constructions using high-performance glass fiber; characterize the 3D structures with respect to thickness, fiber volume fraction and mechanical behavior; and optimize the resin application and curing parameters for each type of 3D woven fabric in order to achieve the desired mechanical properties.

## 2. Materials and Methods

### 2.1. Materials

Continuous multifilament glass tows (Saint-Gobain ADFORS Ltd., Litomyšl, Czech Republic) with a linear density of 600 tex were used to prepare various 3D woven fabrics. Araldite^®^ LY 556 epoxy resin (SPOLCHEMIE in Ústí nad Labem, Czech Republic) was used as a matrix for composite preparation. It is an amine-cured laminating system without reactive diluents that shows excellent flexibility and high reactivity. It is a clear, pale yellow liquid in visual appearance. The density of this resin is 1.15–1.20 g/cm^3^ with a viscosity of 10,000–12,000 cps at 25 °C. A hardener (CHS-HARDENER P11) was mixed with epoxy resin as per the manufacturer (SPOLCHEMIE in Ústí nad Labem, Czech Republic) guidelines with various ratios (8%, 10%, 12% and 14% of the matrix weight) and stirred well for uniform mixing.

### 2.2. Methods

#### 2.2.1. Three-Dimensional Fabric Manufacturing

**Figure 1:**
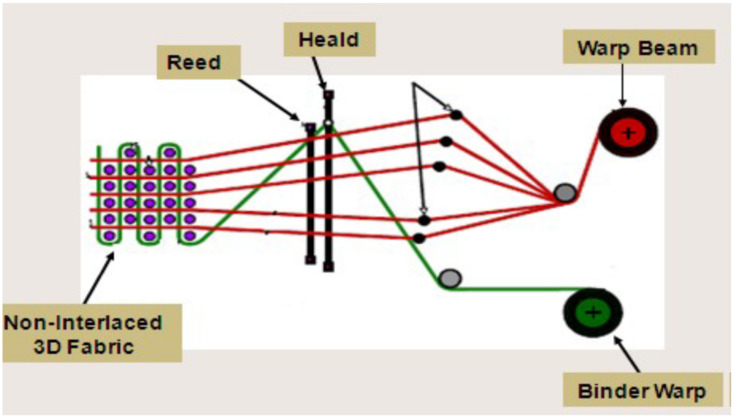
Principle of 3D weaving on a 2D weaving machine.

There are various methods of manufacturing 3D woven fabrics according to the fabric structure. The CCI sample weaving machine was used for this purpose. Some modifications in the conventional loom were made in order to weave a 3D fabric with a 2D loom. A separate negative let-off arrangement was made behind the loom to hold the binder beam while preparing 3D woven fabrics [26,27]. Three types of solid 3D woven structures, viz. orthogonal, angle interlock and warp interlock fabrics, were prepared on the same machine. The beams required to feed this loom were prepared on the CCI sample warping machine. The principle of 3D weaving on a 2D weaving machine is shown in Figure 1.

The weaving machine parameters are given in Table 1.

**Figure 2:**
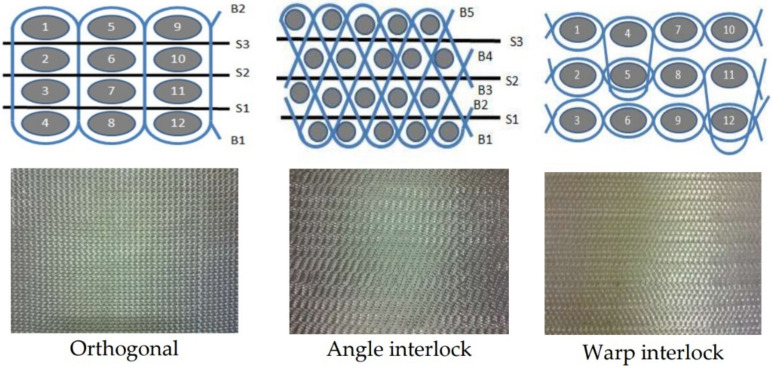
Schematics of the 3D woven structures.

The schematic arrangement of constituent filaments/tows in the 3D woven fabrics is shown in Figure 2.

#### 2.2.2. Composite Manufacturing

##### Compression Molding

All composite samples were prepared using the compression molding technique. LY556 epoxy resin and hardener (CHS-HARDENER P11) from SPOLCHEMIE in Ústí nad Labem, Czech Republic, were used as a matrix component for all the various 3D fabric types. The resin and hardener were mixed and stirred thoroughly as per the manufacturer’s guidelines. The principal advantage of compression molding is its ability to produce parts with a complex geometry in a short period. A SANTECH compression molding machine (Model SMC/DMC/FRP, Gurgaon, India) was used to prepare the composites. It operates at a high speed and with a high degree of precision with computer control. The slide moves with eight-point gibs with a lubrication arrangement that is a specially designed square gib construction that provides accurate guiding of the moving platen/slide with extra stability to resist any deflection under different load conditions. The structure of the press is monolithic or variable depending on requirements. The parameters set on the machine while preparing composites are mentioned below:

Machine used: SANTECH compression molding machine;

Curing time: 900 s;

Molding pressure: 6 bar;

Curing pressure: 12 bar;

Curing temperature: 120 °C;

Hardener/epoxy ratio: 1:10.

##### Specifications of Composite Samples

The composites were developed with different ratios of reinforcing 3D woven glass fabric and matrix (including resin and hardener). The final oven-dry weight of the composite samples was determined after curing and the add-on (%) of the matrix was calculated based on the oven-dry weight of the reinforcing glass fabric.

(1) $$\mathrm{Add}-\mathrm{on}\;\left(\%\right)\;\mathrm{of}\;\mathrm{matrix}=\frac{100\times \left(\mathrm{Oven}\;\mathrm{dry}\;\mathrm{weight}\;\mathrm{of}\;\mathrm{composite}\;-\;\mathrm{Oven}\;\mathrm{dry}\;\mathrm{weight}\;\mathrm{of}\;\mathrm{fabric}\right)}{\mathrm{Oven}\;\mathrm{dry}\;\mathrm{weight}\;\mathrm{of}\;\mathrm{reinforcing}\;\mathrm{fabric}}$$

The specifications of the composite samples prepared are given in Table 2.

#### 2.2.3. Testing

##### Tensile Testing

**Figure 3:**
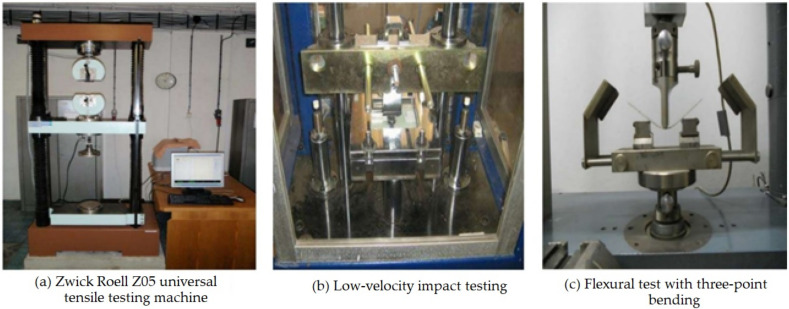
Mechanical testing of the composite samples.

To measure the tensile properties, composite samples were cut in the size of 200 mm × 2.50 mm and the test was carried out on a universal testing machine Z05 (Zwick/Roell, Ulm, Germany) as per the ASTM-D 3039 standard [28]. The testing device works on the principle of a constant rate of elongation (CRE), which was set to 2 mm/min. A Vernier caliper was used before testing to measure the thickness of each specimen. The effective gauge length of the test samples was set as 100 mm. The load cell applied was 1000 kN [29,30]. The maximum load of the specimens was noted before fracture or failure. By monitoring the strain and load of the specimens, the stress–strain response was plotted. From this plot, the tensile modulus and ultimate tensile strength were calculated. For each sample, 30 measurements were carried out. The mean and standard deviation were calculated with a coefficient of variation (CV% < 5%). A Zwick/Roell Z05 Universal testing machine is shown in Figure 3a.

##### Low-Velocity Impact Testing (Gardner Impact Test)

The Gardner impact test was carried out on all the composite samples to estimate the amount of impact energy absorbed by using ASTM D5420-21 [31]. The drop weight impact tester (Model: HIT230F, Zwick Roell Group, Ulm, Germany) was used to measure the energy absorbed by the samples. A hemispherical indenter at a constant rate of 5 m/s was used to carry out this test. A predetermined weight (1.04 kg) and height were determined accordingly for a 10 J impact energy. The weight was allowed to strike on composite specimens, which were supported on a horizontal platform with clamps on the edges. This test was used to measure the damage resistance of composite samples against a dropped weight impact event [32,33]. A flat rectangular sample of dimension 15 cm × 10 cm was subjected to concentrated impact using a drop weight device. The potential energy of the impactor was pre-calculated using the weight and height of the impactor. Damage was imparted through an out-of-plane, concentrated impact that was perpendicular to the plane of the sample. For each sample, 30 measurements were carried out. The peak load and total energy absorbed by the sample was obtained from the test results. The mean and standard deviation were calculated. The device for the impact test is shown in Figure 3b.

The maximum energy of 10 J was applied on the samples through the dropped weight impact tester, where the absorbed energy was calculated as follows:

Impact energy absorbed

(*E*) = *M* × *g* × *h* (2)

where *M*—mass of the dropped weight, *g*-acceleration due to gravity and *h*—height of the fall of the impactor.

Thirty measurements were carried out for each sample and the mean was reported with a coefficient of variation (CV% < 5%). All results were considered for the calculation of the mean. In case some results were outside the variation (CV > 5%), the measurements were repeated.

##### Flexural Test

The flexural properties of composite samples were evaluated using the three-point bending test according to the standard test method ASTM-D7264 [34]. The machine speed was set to 1 mm/min and a force was continuously applied on the specimen until it fractured or the value of the force reduced to 40% of the maximum force. The Zwick/Roell universal testing device (Zwick Roell Group, Ulm, Germany) was used by changing the clamps. It measures the flexural stiffness and strength properties of polymer matrix composites. A specimen of rectangular shape having dimensions 120 mm × 13 mm was supported at the ends and deflected at the center point [35,36]. As the force was applied on the specimen and it started deflecting from the center, its deflection and force were measured and recorded until failure occurred or the maximum force was reduced to 40%. For each sample, 30 measurements were carried out. The mean and standard deviation were calculated with a coefficient of variation (CV% < 5%). The principle of three-point bending is shown in Figure 3c.

Flexural strength is defined as the maximum stress in the outermost fiber while flexural modulus is calculated from the slope of the stress vs. strain curve. A gauge length/support span of 80 mm, deformation rate of 1 mm/min and load cell of 5 kN was applied as per ASTM-D7264 [35,36]. The flexural properties were calculated using Equations (3)–(5).

Flexural stress

(3) $$\sigma_{f}=\frac{3PL}{2bd^{2}}$$

Flexural strain

(4) $$\epsilon_{f}=\frac{6Dd}{L^{2}}$$

Flexural modulus

(5) $$E_{f}=\frac{\sigma_{f}}{\epsilon_{f}}$$

where

$\sigma_{f}$—stress in outer fibers at the midpoint (MPa);
$\epsilon_{f}$—strain in the outer surface (mm/mm);
$E_{f}$—flexural modulus of elasticity (MPa);
*P*—load at a given point on the load deflection curve (N);
*L*—support span (mm);
*b*—width of test beam (mm);
*d*—depth of tested beam (mm);
*D*—maximum deflection of the center of the beam (mm).

## 3. Results and Discussion

### 3.1. Tensile Properties of Composites

The tensile properties of the 3D woven composite samples were evaluated and compared. The effects of the processing and curing parameters were studied.

#### 3.1.1. Effect of Add-On Percentage on Tensile Properties of 3D Woven Composites

**Figure 4:**
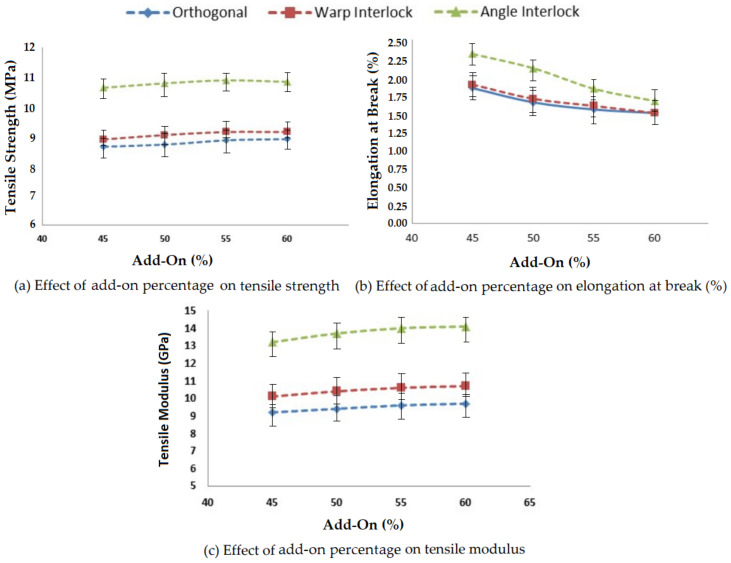
Effect of add-on percentage on the tensile properties of 3D woven composites.

Add-on percentage is defined as the final weight fraction of the matrix in a composite system. It significantly affects the tensile properties of reinforced composites. Four different levels of matrix add-on percentage were selected within a reasonably practical range (45–60%). The developed 3D woven glass fabric reinforced composite samples were tested on Instron tensile tester as per the ASTM-D 3039 standard. The results are shown in Figure 4.

As the matrix add-on percentage increased, the tensile strength and tensile modulus increased due to the better interface between the resin and 3D woven reinforcement. The elongation at break of the composite samples decreased as the add-on percentage increased. This was due to better bonding between the resin and reinforcement. There was a steady increase in the tensile strength and modulus when the add-on percentage was increased from 45% to 55%. However, when the add-on percentage was increased beyond 55%, there was no further improvement in tensile properties. Thus, a 55% matrix and 45% fiber could be treated as optimal for 3D woven glass–epoxy composites.

The tensile strength, elongation at break and tensile modulus were maximum for the angle interlock structure and the other two structures showed similar tensile properties. The geometry of the angle interlock fabric was responsible for the higher load bearing. Further, the angular disposition of the binder yarn enabled higher elongation at break compared to the other 3D woven structures. The results showed significance at the 95% confidence interval. The CV was lower than 5%.

#### 3.1.2. Effect of the Amount of Hardener on Tensile Properties

**Figure 5:**
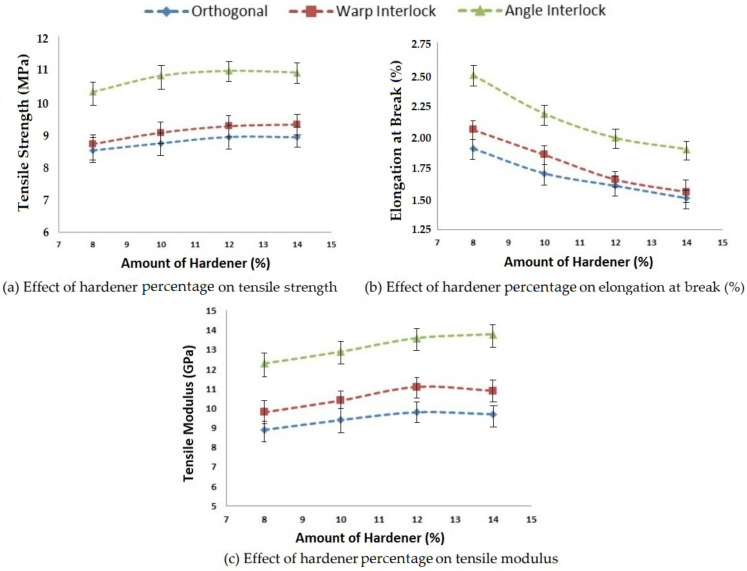
Effect of the amount of hardener percentage on the tensile properties of 3D woven composites.

Tensile properties, such as tensile strength, elongation at break and tensile modulus, are also affected by changing the amount of hardener added with resin during composite preparation. In this study, four different amounts of hardener were selected as per the guidelines of the supplier. The composite samples with 3D woven fabric reinforcement were tested in order to study the influence of the hardener on the tensile properties. The results obtained for different 3D glass woven fabric reinforced epoxy composites are shown in Figure 5.

**Figure 6:**
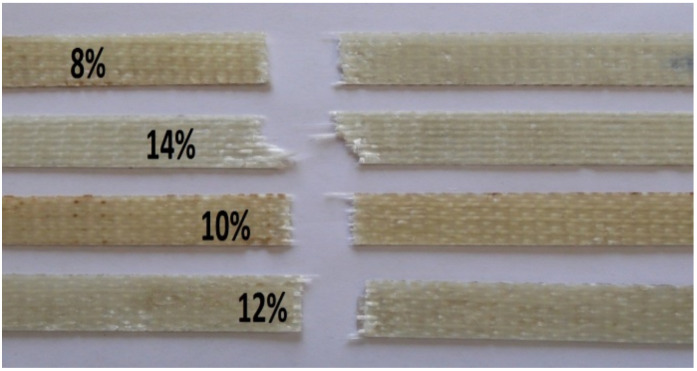
Nature of tensile failure in 3D woven glass–epoxy composites with different hardener percentages.

An increase in hardener percentage increased the tensile strength as well as the modulus. There was a consistent increment up to the addition of 12% hardener in the matrix. However, thereafter, the strength and modulus remained almost constant. There was no further increase in strength or modulus when the amount of hardener was increased to 14%. Therefore, 12% hardener could be considered optimal for 3D woven glass–epoxy composites. Angle interlock structure-based epoxy composites exhibited superior tensile properties with varying amounts of hardener (%) compared to orthogonal and warp interlock fabrics. The composite samples with different hardener percentages after tensile testing are shown in Figure 6.

As can be observed, the samples with 8%, 10% and 12% hardener showed a straight-line failure after tensile testing. This was a good indication of uniform load bearing by all the constituent filaments/tows in the testing direction. Such behavior also indicated a homogenous impregnation of the fiber and a good interface with the matrix. However, with 14% hardener, there was an angled line of failure. This indicated that the higher hardener percentage (more than 12%) led to nonuniformity of resin impregnation and nonuniform load bearing by the constituent reinforcing elements in the 3D woven glass–epoxy composite.

#### 3.1.3. Effect of Curing Temperature on Tensile Properties

**Figure 7:**
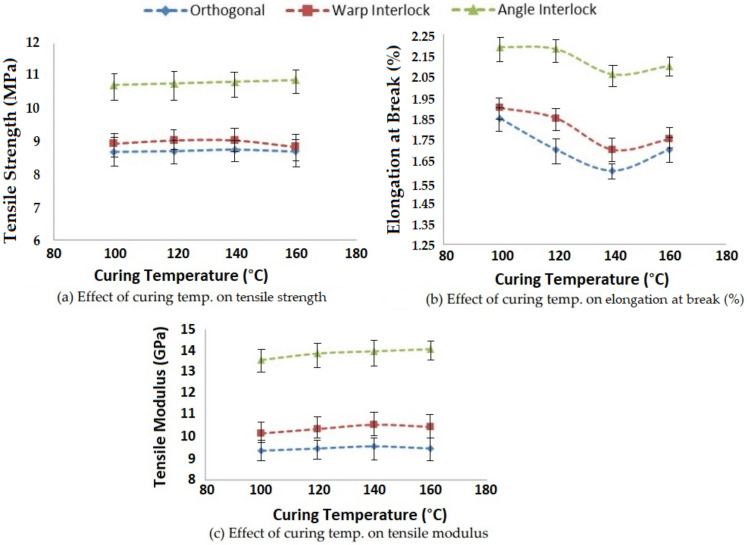
Effect of curing temperature on the tensile properties of 3D woven composites.

Curing is an important process in composite preparation and the parameters, e.g., curing time and temperature, of the process affect the tensile properties of composites. Curing temperature also affects the viscosity of the resin. On increasing the temperature, the viscosity of resin reduces. Viscosity is directly associated with resin impregnation into reinforcement, which ultimately affects the tensile properties, such as the strength, elongation at break and tensile modulus. The effect of curing temperature between 100 °C and 160 °C on the tensile performance of 3D woven polymer composites is shown in Figure 7.

As the curing temperature increased, the viscosity of resin decreased, which facilitated easier resin impregnation into the fibrous structure (3D woven fabric). The resin was able to bind the reinforcing 3D structure more effectively. This ultimately increased the tensile strength and modulus to an optimal value. This trend was observed to be almost similar for all three types of 3D woven fabric reinforced composites. A curing temperature of about 140 °C seemed to be optimal for glass–epoxy composites. The results showed significance at the 95% confidence interval. The CV was lower than 5%.

#### 3.1.4. Effect of Curing Time on Tensile Properties

**Figure 8:**
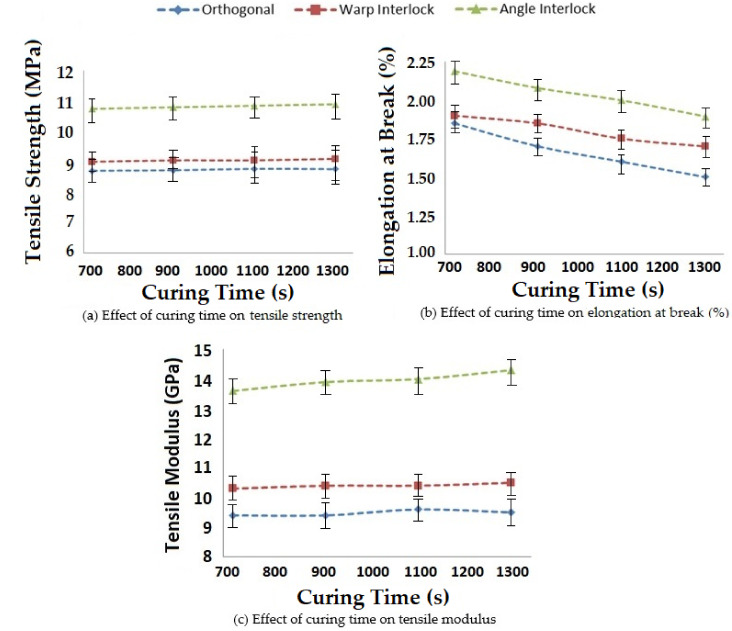
Effect of curing time on the tensile properties of 3D woven composites.

The influence of curing time on the tensile properties of 3D woven polymer composites was studied. The results obtained for curing times between 720 s and 1260 s are shown in Figure 8. This range was selected based on the recommendation of the resin supplier.

The effect of curing time on the tensile properties of the composites seemed to follow a similar trend to the curing temperature. As the curing time increased, the tensile strength and tensile modulus increased for all three types of glass–epoxy composites using 3D woven structures. With increasing time, there was better impregnation of resin, which was responsible for the improved tensile properties. However, excessive curing time might lead to degradation of the resin. As usual, the angle interlock structure showed superior tensile properties based on the internal geometry of the fabric.

#### 3.1.5. Effect of Molding Pressure on Tensile Properties

**Figure 9:**
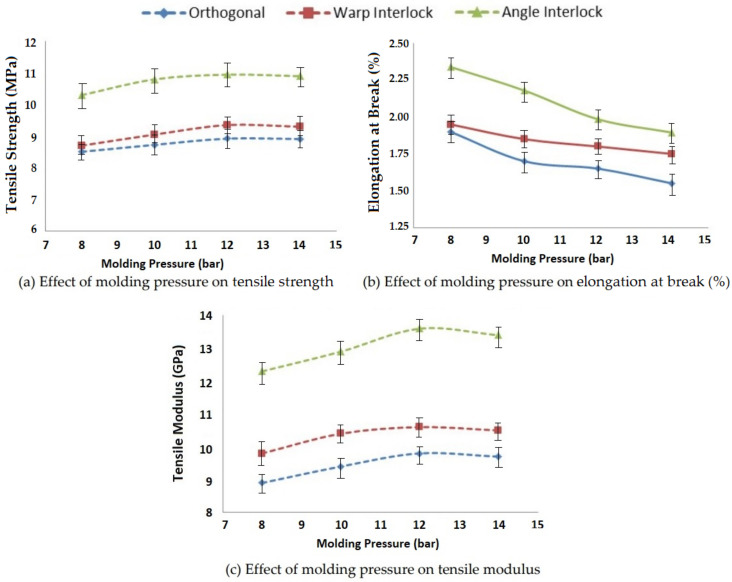
Effect of molding pressure on the tensile properties of 3D woven composites.

Pressure is applied in the mold when the curing of a composite is carried out. That pressure directly affects the resin impregnation into the reinforcement structure and, consequently, the resin impregnation affects the tensile properties, such as tensile strength, elongation at break and tensile modulus. Four levels of molding pressure, i.e., 8, 10, 12 and 14 bar, were selected, and the obtained results are shown in Figure 9.

Increasing the molding pressure helped with providing better penetration of the resin into the fabric, resulting in higher tensile strength and modulus, as is visible from Figure 9. This trend was visible up to a molding pressure of 12 bar. However, a further increase in molding pressure did not seem to increase the strength and modulus.

With the increase in pressure, there was more consolidation of the fibers and resin in the composite, resulting in a stronger interface. Thus, the elongation at break decreased. This trend was observed for all three types of 3D woven reinforcement of glass fibers. The results showed significance at the 95% confidence interval. The CV was lower than 5%.

**Figure 10:**
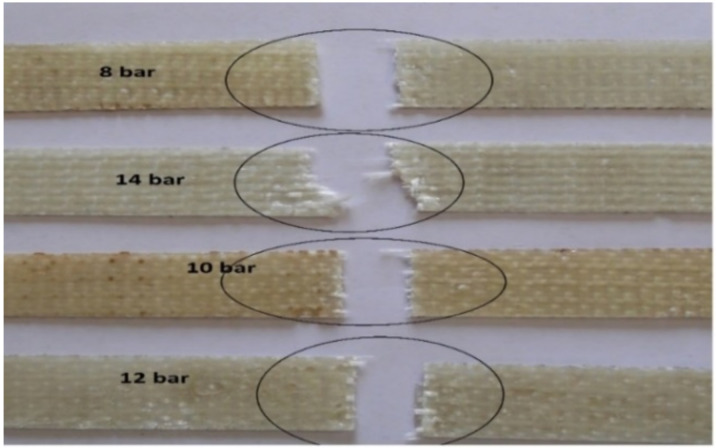
Nature of tensile failure in the 3D woven glass–epoxy composites manufactured with different molding pressures.

The 3D woven composite samples developed with different molding pressures and that fractured after the tensile test are shown in Figure 10.

**Figure 11:**
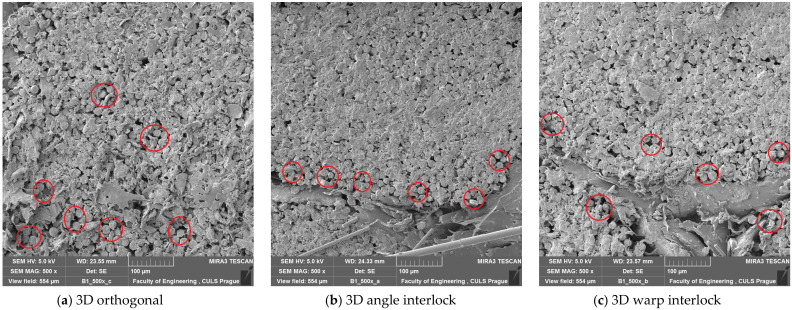
SEM images of tensile tested 3D woven composite samples prepared at 12 bar molding pressure.

The nature of the failure was uniform till a pressure of 12 bar, as is visible from Figure 10. Therefore, an optimal pressure of 12 bar can be used during molding for 3D woven glass–epoxy composites. Some SEM images of tensile tested samples prepared at 12 bar molding pressure are shown in Figure 11. The fractured surface showed sharp edges and catastrophic failure of the fibers. The fiber rupture (in red circles) indicated that they uniformly bore the load from the resin.

### 3.2. Impact Properties of Composites

The impact properties of the different 3D woven composite samples were evaluated and compared. The effects of resin processing and curing parameters were studied in detail.

#### 3.2.1. Effect of Matrix Add-On Percentage on Impact Properties

**Figure 12:**
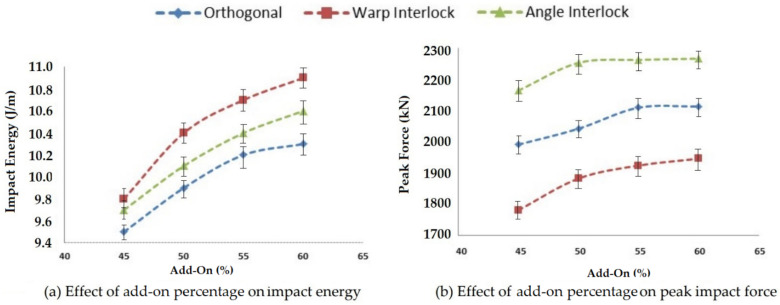
Effect of add-on percentage on the impact properties of 3D woven composites.

Matrix add-on percentage affects the impact properties (impact energy, peak force and deformation). Impact testing was carried out using ASTM D5420-21 for the developed composite samples with different 3D woven reinforcements impregnated with epoxy resin at various levels of matrix add-on percentage, i.e., 45, 50, 55 and 60%. The obtained results are compared and shown in Figure 12.

With an increasing level of matrix add-on percentage, all the composite samples with different reinforcement structures followed a similar trend. The impact energy and the peak impact force increased to an optimal value at 55% add-on and after that, there was no significant increase.

The highest impact energy was observed for warp interlock fabric composite samples and lowest for orthogonal structure reinforced samples. This may have been due to the interlaminar integrated structure of the warp interlock fabric, where the Z-direction yarn showed maximum total deflection, leading to the maximum impact energy (area under the impact force and deflection curve). In the case of the orthogonal structure, the un-crimped orthogonal yarns/tows did not facilitate much high energy absorption.

The peak impact force increased with the increase of matrix add-on percentage. The trend was similar for all three structures (orthogonal, angle interlock and warp interlock) to a certain level. On the further increase of matrix add-on, there was no penetration inside the reinforcement structure. This optimal level was attained at about 55% of resin add-on.

The highest impact force was observed for the angle interlock structure and the lowest value was for the warp interlock structure. In the angle interlock fabric, the consolidation of the different layers was possible to the maximum extent. The angular disposition of the Z yarn, which enabled maximum tensile strength and modulus, also helped in providing the maximum impact strength. The warp interlock structure did not integrate all layers into a single interlacement. It bound the layers step by step. Thus, the load bearing was not the maximum, but energy absorption was facilitated. That is why the warp interlock fabric-based composite showed maximum impact energy absorption. The orthogonal fabric reinforced composite showed an intermediate peak impact force due to the lowest deformation pertaining to non-crimped constituent yarns. The results showed significance at the 95% confidence interval. The CV was lower than 5%.

#### 3.2.2. Effect of the Amount of Hardener on Impact Properties

**Figure 13:**
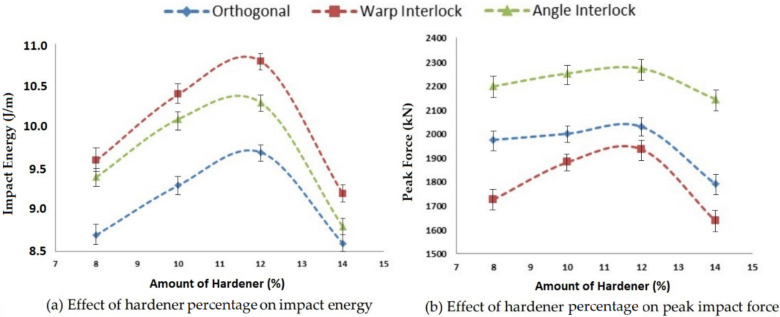
Effect of the amount of hardener percentage on the impact properties of 3D woven composites.

A hardener is used for the hardening of a matrix system. The hardening rate of a matrix is affected by the amount of hardener used in the composite preparation. This ultimately affects the impact properties of composites, such as impact energy, peak impact force and deformation. For analyzing the effect of the hardener percentage, four different levels, i.e., 8%, 10%, 12% and 14%, were used, and the samples were tested as per standard ASTM D5420-21. The results are shown in Figure 13.

On increasing the amount of hardener, the impact energy and peak impact force increased for all three types of 3D woven composite structures up to the optimal level. This was due to the better hardening rate and strengthening of the interface with resin. However, beyond a 12% hardener amount, the impact energy, as well as peak impact force, decreased, which may have been due to excessive hardening of the matrix. The reinforcement structure could not be impregnated uniformly and thus was not able to bind the structure effectively.

The highest impact energy was observed for the warp interlock composite and the lowest for orthogonal fabric-based samples. This could be attributed to the energy absorption capacity, which depended on the disposition of binder yarns/tows (Z yarns). The highest deformation occurred in the warp interlock structure and the lowest deformation occurred in the orthogonal structure. The angle interlock fabric composites exhibited the highest impact strength pertaining to load-bearing capacity, resulting from the angular interlacement and maximum consolidation of the reinforcement fabric. In contrast, the warp interlock fabric composite shows the lowest peak force level due to the layer-by-layer binding, which does not allow for maximum consolidation. However, a maximum displacement/post-impact deformation helped with the highest impact energy absorption in this case.

#### 3.2.3. Effect of Curing Temperature on Impact Properties

**Figure 14:**
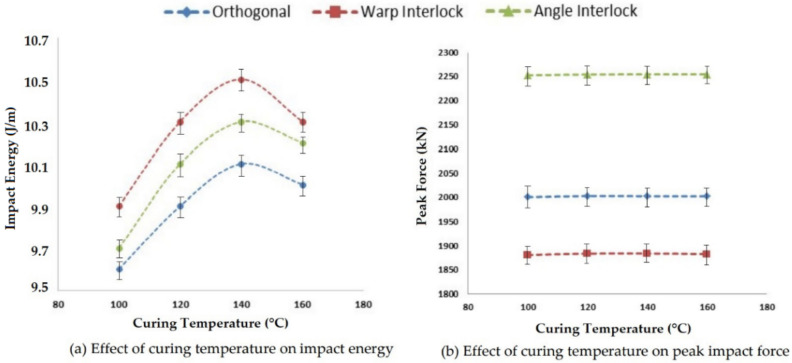
Effect of curing temperature on the impact properties of 3D woven composites.

Curing is an important process of composite preparation and the variables, such as curing time and temperature, of the process affect the impact properties of a composite. Curing temperature influences the resin impregnation into reinforcement structure, which will affect the impact properties, such as impact energy, peak impact force and deformation. The curing temperature was varied between 100 °C and 160 °C in four steps. The effect of curing temperature on impact energy and peak impact force is shown in Figure 14.

As the curing temperature was increased, the impact energy also increased for all 3D woven composites. The increase was within a very narrow range (9.6 J/m–10.5 J/m). The increase in impact energy was due to better impregnation of the resin as the viscosity of the resin reduced with increased temperature. Beyond a curing temperature of 140 °C, the resin started degrading, which reduced the adhesion properties of the resin. Thus, deterioration of impact energy was observed.

The highest impact energy was observed for warp interlock fabric composites and was the lowest for orthogonal structure composites. The peak impact force was observed to be at a maximum for angle interlock composites and at a minimum for warp interlock fabric composites. The peak impact force was not very dependent on the curing temperature. A sufficient temperature was required, at which, the resin would bind the reinforcement assembly. The optimal temperature for curing 3D woven fabric glass–epoxy composites was found to be 140 °C. The results showed significance at the 95% confidence interval. The CV was lower than 5%.

#### 3.2.4. Effect of Curing Time on Impact Properties

**Figure 15:**
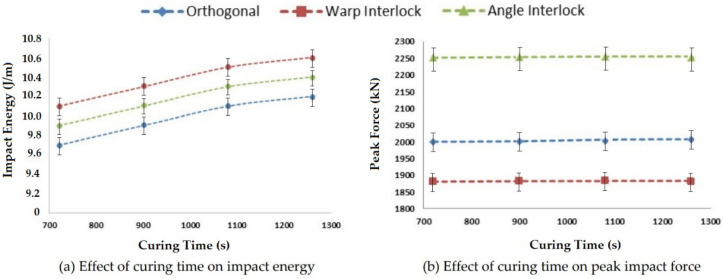
Effect of curing time on the impact properties of 3D woven composites.

The curing time is also an important parameter that influences the impact performance of polymer composites. The variation of curing time between 720–1260 s was studied and its influence on the impact properties is shown in Figure 15.

The curing time showed a similar trend to curing temperature. However, the impact energy kept increasing beyond the curing time of 1260 s. The peak impact force remained unchanged with the variation in curing time. A higher curing time might cause degradation of the resin.

#### 3.2.5. Effect of Molding Pressure on Impact Properties

**Figure 16:**
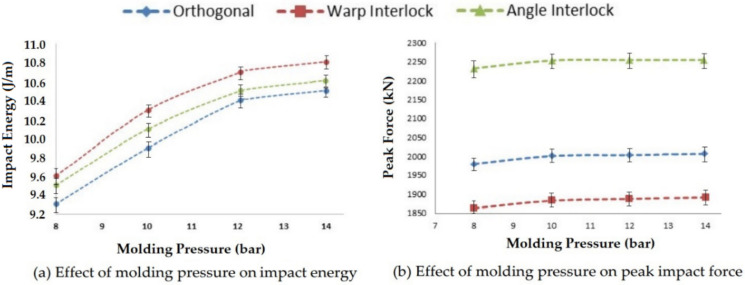
Effect of molding pressure on the impact properties of 3D woven composites.

When pressure is applied into the mold during curing, it directly affects the resin impregnation into the reinforcement, where the resin impregnation affects the impact properties, such as impact energy, peak impact force and deformation. Four levels of molding pressure, i.e., 8, 10, 12 and 14 bar, were applied, and the results are shown in Figure 16.

**Figure 17:**
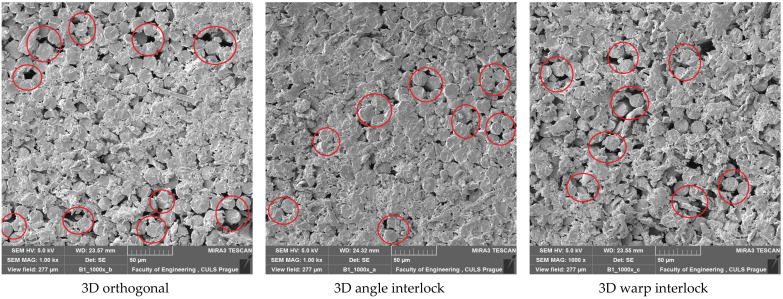
SEM images of impact-tested 3D woven composite samples prepared at 12 bar molding pressure.

On increasing the molding pressure, the impact energy and peak impact force increased for all three types of 3D woven composite structures. The molding pressure increased the resin impregnation in the reinforcement, which improved the binding between the resin and reinforcement and ultimately improved the impact performance. After the optimal pressure of 12 bar, there was not much increase in impact energy and peak impact force. The impact energy was found to be highest for the warp interlock structure composite and at a minimum for the orthogonal 3D woven fabric glass–epoxy composite. This was due to the interlacement pattern. Further, the peak impact force was highest for the angle interlock structure reinforced composite and at a minimum for the warp interlock structure composite, as observed in previous results in this study. SEM images of the impact-tested composite samples prepared at 12 bar molding pressure are shown in Figure 17. The images show sharp edges, which indicate catastrophic fiber breakage. The fiber rupture (in red circles) indicated that they bore a uniform load from the resin.

### 3.3. Flexural Properties of Composites

#### 3.3.1. Effect of Matrix Add-On Percentage on Flexural Properties

**Figure 18:**
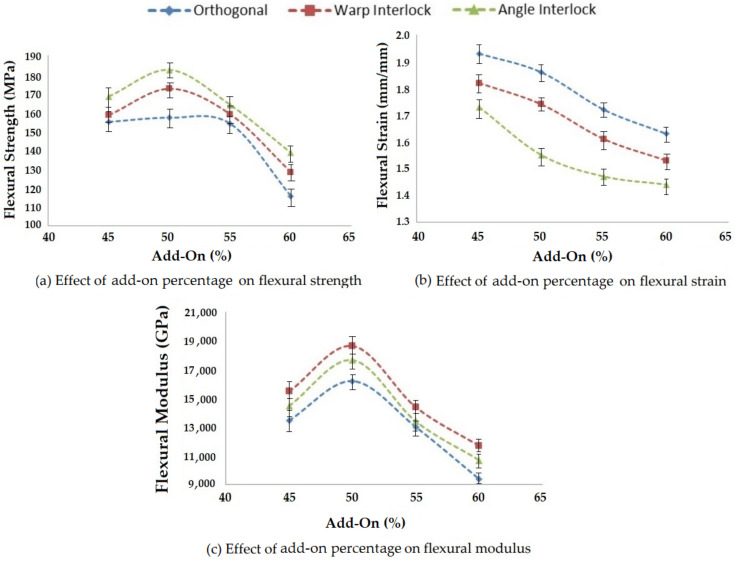
Effect of add-on percentage on the flexural properties of 3D woven composites.

The add-on percentage of the matrix affects the bending properties of composites. Four different levels of matrix add-on percentage were studied and the prepared composite samples with different 3D woven fabric reinforcement were tested in terms of three-point bending properties as per ASTM-D7264. The results are shown in Figure 18.

As the matrix add-on percentage increased, the flexural strength increased for all three types of 3D woven reinforcement structures due to the increase of the binding element (resin) in the composite. However, after an optimum value of add-on (about 50%), the strength decreased, which may have been due to an insufficient fraction of fiber element in the composite. The flexural strain decreased with an increase of matrix add-on percentage for all three fabric structures. This was due to the higher restriction to deformation of fiber components when an excessive amount of resin was applied.

The flexural modulus followed a similar trend to that of flexural strength for all three types of 3D woven composite structures. There was an increase in flexural modulus while using up to 50% matrix add-on. Beyond this limit, a steady decrease in flexural modulus was observed.

Flexural strength was observed to be at a maximum for the angle interlock structure due to better resin impregnation. The angular arrangement of Z yarns consolidated the structure and increased the strength in all deformation modes. The lowest flexural strength was observed for orthogonal structure due to the perpendicular/uncrimped Z yarns, which provide no interlacement.

The flexural strain was at a maximum for the orthogonal structure, as the Z-direction yarn was at 90° and resulted in high strain.

The flexural modulus was at a maximum for the warp interlock fabric composites due to the optimal flexural stress/strength and strain. The layer-by-layer bindings in the warp interlock fabric helped resist the bending deformation. A 50% matrix add-on was optimal for flexural strength, as well as modulus. The results showed significance at the 95% confidence interval. The CV was lower than 5%.

#### 3.3.2. Effect of the Amount of Hardener on Flexural Properties

**Figure 19:**
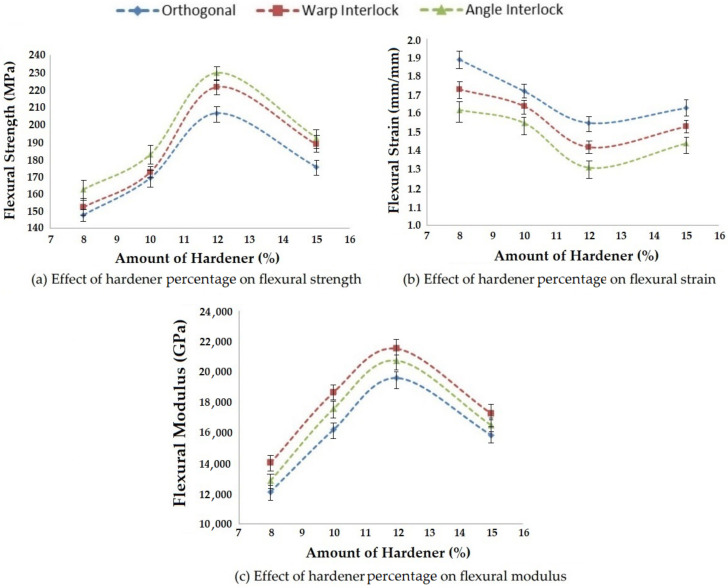
Effect of the amount of hardener on the flexural properties of 3D woven composites.

Flexural properties, such as flexural strength, flexural strain and flexural modulus, are also affected by changing the amount of hardener added with resin during composite preparation. In this study, four levels of hardener percentage were selected and the effects on bending properties were observed. The results are shown in Figure 19.

As the amount of hardener increased, the flexural strength and modulus also increased due to the increase in the adhesion properties of the resin. An optimal value of flexural strength and modulus was achieved at 12% hardener. With the further increase of the hardener amount, there was a decrease in flexural properties due to excessive hardening of the resin before curing. This trend was observed for all three types of 3D woven reinforcements. The flexural strain decreased until an optimum amount of hardener (12%) and then increased. This could be attributed to the hardening of resin before curing and improper adhesion with the reinforcement. As usual, the angle interlock fabric composite showed the highest flexural strength, followed by the warp interlock fabric composite. The orthogonal glass fabric epoxy composite showed the minimum flexural strength among all the 3D woven structures. The trends of flexural strain were opposite to that of flexural strength. The orthogonal fabric composites exhibited the maximum strain due to non-interlaced Z yarns. The results showed significance at the 95% confidence interval. The CV was lower than 5%.

#### 3.3.3. Effect of Curing Temperature on Flexural Properties

**Figure 20:**
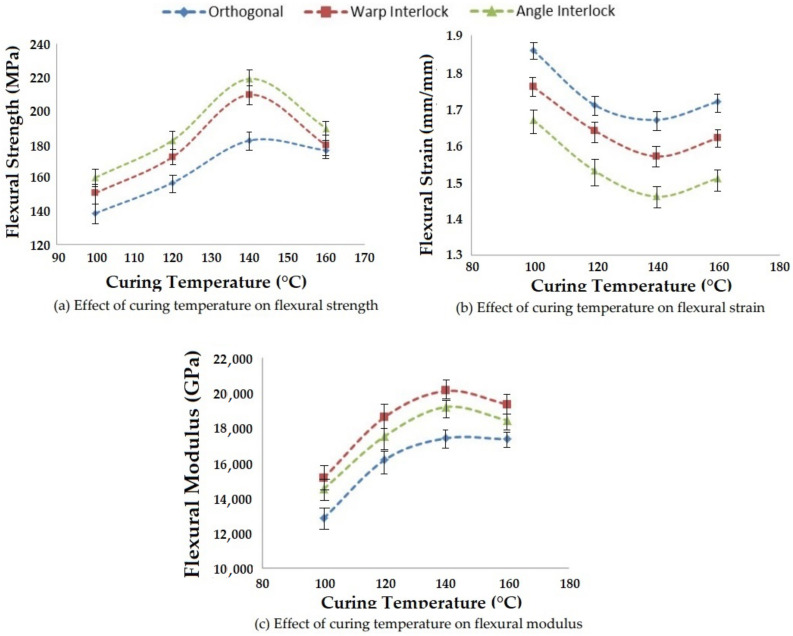
Effect of curing temperature on the flexural properties of 3D woven composites.

Curing process parameters, e.g., temperature and time, significantly affect the bending properties of composites. By increasing the curing temperature, the viscosity of the resin reduces and changes the impregnation efficiency. This consequently affects the flexural strength, flexural strain and flexural modulus. The influence of different curing temperatures on flexural properties of 3D woven fabric glass–epoxy reinforced composites is shown in Figure 20.

As the curing temperature increased, the viscosity of resin decreased, which increased the resin impregnation into the structure and helped with binding the reinforcement structure more effectively. This increased the flexural strength and modulus. Such a trend was observed for all the three 3D woven reinforcements up to a curing temperature of 140 °C. However, after the optimal level, the flexural properties started decreasing, probably due to the degradation of the resin.

The flexural strain consistently decreased when the curing temperature was increased from 100 °C to 140 °C. However, beyond this temperature, there was an increase in flexural strain. This trend was similar for all three types of 3D woven fabric structures. The increase in flexural strain, along with the decrease of flexural stress, resulted in an overall reduction in flexural modulus. The results showed significance at the 95% confidence interval. The CV was lower than 5%.

#### 3.3.4. Effect of Curing Time on Flexural Properties

**Figure 21:**
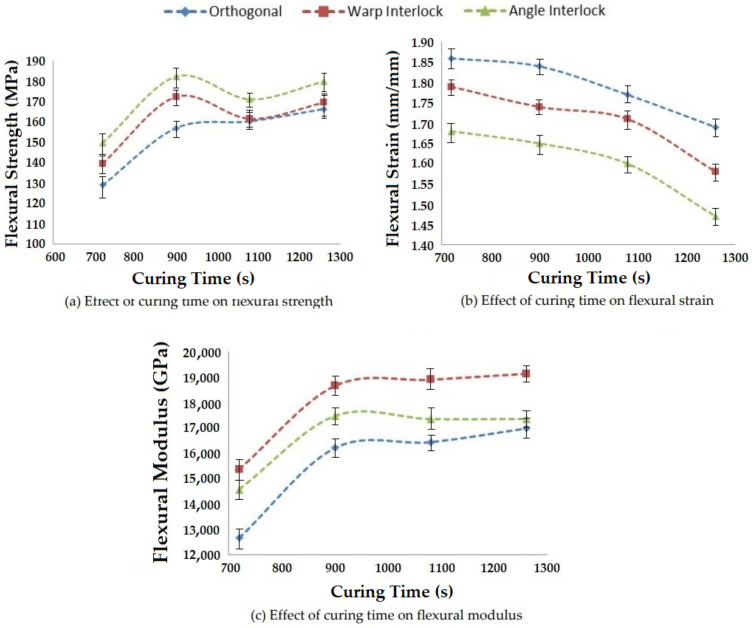
Effect of curing time on the flexural properties of 3D woven composites.

The duration of curing was changed in order to study its influence on the bending properties of polymer composites reinforced with 3D woven fabrics. The results are shown in Figure 21.

A curing time of 900 s was found to be optimal for all types of 3D woven glass fabric reinforced epoxy composites. The maximum flexural strength and modulus were obtained with this curing duration. When the curing time was increased further, there was no improvement in flexural properties. The flexural strain systematically decreased with increasing curing time. Angle interlock fabric-based composites showed maximum flexural strength and minimum flexural strain. However, the warp interlock fabric composites resulted in maximum flexural modulus due to the optimal values of flexural stress and strain. This was again attributed to the interlacement pattern and layer-by-layer binding in the reinforcement structure.

#### 3.3.5. Effect of Molding Pressure on Flexural Properties

**Figure 22:**
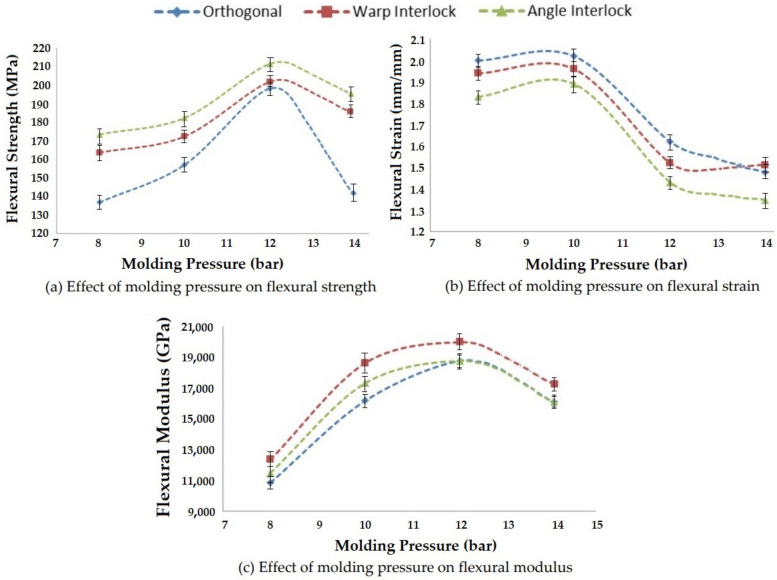
Effect of molding pressure on the flexural properties of 3D woven composites.

The molding pressure directly affected the resin impregnation into the reinforcement and thereby the flexural properties, such as flexural strength, flexural strain and flexural modulus. The influence of molding pressure (at 8, 10, 12 and 14 bar) on the flexural properties of 3D woven glass–epoxy composites was studied. The results are shown in Figure 22.

On increasing the molding pressure, the flexural strength and flexural modulus increased for all three types of 3D woven composite structures. This was due to the improved resin impregnation through the reinforcement, which increased the binding between resin and reinforcing fibers. A molding pressure of 12 bar resulted in the highest flexural strength and flexural modulus. The results showed significance at the 95% confidence interval. The CV was lower than 5%.

The flexural modulus was observed to be the highest for the warp interlock structure epoxy composite and minimum for orthogonal 3D structure composite. In all three types of 3D woven fabric glass–epoxy reinforced composites, the flexural strength and modulus decreased when the molding pressure was increased beyond 12 bar.

## 4. Conclusions

Three-dimensional woven glass fabrics with three different architectures, i.e., orthogonal, angle interlocked and warp interlocked structures were developed on a CCI sample weaving machine with an arrangement with an extra beam and a negative let-off motion. The matrix add-on (%), amount of hardener (%), curing temperature, curing time and molding pressure were selected as important parameters that affect resin infusion in the 3D woven structure and, thus, the ultimate mechanical properties of polymer composites.

The tensile strength, elongation at break and tensile modulus were found to be at a maximum for the angle interlock structure among the various 3D woven composites. As the matrix add-on percentage increased, the tensile strength and tensile modulus increased due to the better interface between the resin and 3D woven reinforcement. The elongation at break decreased as the add-on percentage increased. This was due to better bonding between the resin and reinforcement. A structure with 55% matrix and 45% fiber can be treated as optimal for 3D woven glass–epoxy polymer composites. A 12% addition of hardener (based on weight of the matrix) was found to be optimal for 3D glass woven fabric epoxy composites. A curing temperature of about 140 °C seemed to be optimal for glass–epoxy composites. As the curing time increased, the tensile strength and tensile modulus increased for all three types of glass–epoxy composites. Increasing the molding pressure up to 12 bar helped provide better penetration of the resin, resulting in higher tensile strength and modulus.

The highest impact energy was observed for the warp interlock fabric composites and the lowest for the orthogonal structure reinforced samples. With an increasing level of matrix add-on percentage, all composite samples with different reinforcement structures showed improvement in impact performance up to an optimal add-on value of 55%. Beyond this level, there was no significant improvement in impact properties. A 12% addition of hardener, curing temperature of 140 °C, maximum curing time and 12 bar molding pressure resulted in the highest impact energy and peak impact force.

Flexural strength was observed to be at a maximum for the angle interlock structure composites, whereas the maximum flexural modulus was observed for warp interlock fabric composites due to optimal flexural stress/strength and strain. A 50% add-on of the epoxy matrix resulted in the highest flexural strength and modulus. The optimal conditions for the best flexural performance in 3D woven glass–epoxy polymer composites were 12% hardener, 140 °C curing temperature, 900 s curing time and 12 bar molding pressure.

The most significant parameters were identified as the add-on percentage of the matrix, amount of hardener and molding pressure. These optimized processing parameters can serve as a major guideline for further research and development in glass multifilament-based 3D woven epoxy composites.

## Figures and Tables

**Table 1:** Parameters of the weaving machine.

Sample Type	No. of Beams	No. of Healds	Warps (cm^−1^)	Wefts (cm^−1^)	Fabric Areal Density (g·m^−2^)	Machine Speed (rpm)	Machine Efficiency (%)
3D orthogonal	2	5	10	10	1200 ± 20	600	80
3D angle interlock	2	8	10	10	1200 ± 20	550	73
3D warp interlock	2	6	10	10	1200 ± 20	570	75

**Table 2:** Specification of various composite samples developed with variable parameters.

Variable Parameter	Reinforcement Fabric Type	Weight of Fabric (g)	Weight of Composite (g)	Weight of Matrix (g)	Weight Fraction of Fiber/Fabric in Final Composite
Add-on (%) of matrix					
45	Orthogonal	62.3	93.4	31.1	0.49
	Angle interlock	62.3	93.4	31.1	0.49
	Warp interlock	62.3	93.4	31.1	0.49
50	Orthogonal	63.5	97.2	33.7	0.47
	Angle interlock	63.5	97.2	33.7	0.47
	Warp interlock	63.5	97.2	33.7	0.47
55	Orthogonal	62.3	98.6	36.3	0.45
	Angle interlock	62.3	98.6	36.3	0.45
	Warp interlock	62.3	98.6	36.3	0.45
60	Orthogonal	64.4	103.3	38.9	0.44
	Angle interlock	64.4	103.3	38.9	0.44
	Warp interlock	64.4	103.3	38.9	0.44
Amount of hardener (%)					
8	Orthogonal	64.5	102.4	37.9	0.45
	Angle interlock	64.5	102.4	37.9	0.45
	Warp interlock	64.5	102.4	37.9	0.45
10	Orthogonal	65.4	106.1	40.7	0.44
	Angle interlock	65.4	106.1	40.7	0.44
	Warp interlock	65.4	106.1	40.7	0.44
12	Orthogonal	63.5	105.7	42.2	0.42
	Angle interlock	63.5	105.7	42.2	0.42
	Warp interlock	63.5	105.7	42.2	0.42
14	Orthogonal	62.9	93.4	30.5	0.47
	Angle interlock	62.9	93.4	30.5	0.47
	Warp interlock	62.9	93.4	30.5	0.47
Curing temperature (°C)					
100	Orthogonal	63.4	97.1	33.7	0.47
	Angle interlock	63.4	97.1	33.7	0.47
	Warp interlock	63.4	97.1	33.7	0.47
120	Orthogonal	54.6	86.2	31.6	0.45
	Angle interlock	54.6	86.2	31.6	0.45
	Warp interlock	54.6	86.2	31.6	0.45
140	Orthogonal	62.6	98.9	36.3	0.45
	Angle interlock	62.6	98.9	36.3	0.45
	Warp interlock	62.6	98.9	36.3	0.45
160	Orthogonal	63.5	99.1	35.6	0.46
	Angle interlock	63.5	99.1	35.6	0.46
	Warp interlock	63.5	99.1	35.6	0.46
Curing time (s)					
720	Orthogonal	64.5	99.3	34.8	0.47
	Angle interlock	64.5	99.3	34.8	0.47
	Warp interlock	64.5	99.3	34.8	0.47
900	Orthogonal	63.2	97.2	34	0.47
	Angle interlock	63.2	97.2	34	0.47
	Warp interlock	63.2	97.2	34	0.47
1080	Orthogonal	62.9	94.5	31.6	0.48
	Angle interlock	62.9	94.5	31.6	0.48
	Warp interlock	62.9	94.5	31.6	0.48
1260	Orthogonal	63.3	96.9	33.6	0.47
	Angle interlock	63.3	96.9	33.6	0.47
	Warp interlock	63.3	96.9	33.6	0.47
Molding pressure (bar)					
8	Orthogonal	64.2	98.2	34	0.47
	Angle interlock	64.2	98.2	34	0.47
	Warp interlock	64.2	98.2	34	0.47
10	Orthogonal	63.8	98.5	34.7	0.46
	Angle interlock	63.8	98.5	34.7	0.46
	Warp interlock	63.8	98.5	34.7	0.46
12	Orthogonal	62.7	102.4	39.7	0.43
	Angle interlock	62.7	102.4	39.7	0.43
	Warp interlock	62.7	102.4	39.7	0.43
14	Orthogonal	63.1	99.7	36.6	0.45
	Angle interlock	63.1	99.7	36.6	0.45
	Warp interlock	63.1	99.7	36.6	0.45

## Data Availability

The data presented in this study are available on request from the corresponding author.
